# Editorial: The cognitive era in sports performance: mental fatigue, cognitive training, and psychological ergogenic substances

**DOI:** 10.3389/fpsyg.2025.1640470

**Published:** 2025-06-17

**Authors:** Dalton de Lima-Junior, Thiago Ribeiro Lopes, Leonardo de Sousa Fortes, Fabio Yuzo Nakamura, Samuele Maria Marcora

**Affiliations:** ^1^Department for Life Quality Studies, University of Bologna, Bologna, Italy; ^2^Laboratory of Exercise Physiology at Olympic Center of Training and Research, Department of Physiology, Federal University of São Paulo, São Paulo, SP, Brazil; ^3^Post-Graduate Program in Translational Medicine, Department of Medicine, Paulista School of Medicine, Federal University of São Paulo, São Paulo, SP, Brazil; ^4^Department of Sports, Federal University of Minas Gerais, Belo Horizonte, Minas Gerais, Brazil; ^5^Department of Physical Education and Sports, University of Maia, Maia, Portugal

**Keywords:** sport psychology, perceptual-cognitive skills, mental fatigue, mindfulness, executive function, motor learning

## Unlocking athletic potential through cognitive science

The landscape of elite sports is undergoing a paradigm shift. While physical prowess remains foundational, the next frontier of athletic excellence lies in the mind. A growing body of research demonstrates that cognitive, perceptual, emotional, and motivational factors and interventions—and their underlying neurobiology—play a decisive role in human performance. This editorial synthesizes key findings from recent studies published in *Frontiers in Psychology*, highlighting how psychology and cognitive neuroscience are reshaping training, competition, and recovery in sports.

## The role of psychological interventions

The influence of mental training on athletic performance might no longer be speculative. A randomized controlled trial on Chinese sprinters (Yu et al.) revealed that a 7-week mindfulness program significantly reduced competition anxiety, enhancing somatic and cognitive self-regulation. These findings align with broader evidence that psychological skills training—once considered supplementary—is now essential for peak performance. Nevertheless, despite these advances, performance breakdown under pressure remains a challenge. Interestingly, Thiessen et al. found no direct link between self-reported mental toughness and choking susceptibility, challenging conventional wisdom and underscoring the need for more nuanced models of performance breakdowns in high-stakes scenarios.

## Perceptual-cognitive skills: the hidden edge in sports

Elite performance in fast-paced, open-skill sports (e.g., badminton, fencing) relies on rapid decision-making and spatial awareness. Wu K.-C. et al. demonstrated that working memory capacity distinguishes elite adolescent badminton players from their semi-elite counterparts. Similarly, expert sports officials (Wu Y. et al.) exhibit superior decision-making accuracy and visual efficiency, suggesting that perceptual-cognitive factors are relevant to both athletes and referees.

Spatial cognition has also been linked to sports performance. Feng et al. revealed that athletes in high-spatial-demand sports (e.g., gymnastics, tennis) outperform others in mental rotation tasks, with axial rotation experience mitigating traditional sex differences in spatial ability. These studies suggest that perceptual-cognitive tests and training should be included in the talent identification and training programs of both athletes and referees to improve sports performance.

## Mental fatigue: the silent performance thief in elite sports

While physical fatigue is relatively well-managed in elite sports, mental fatigue (MF) remains an under-addressed performance thief. Bian et al. and de Lima-Junior et al. highlight their potential effects: national-level fencers report heightened MF during elimination rounds, while swimmers experience inflated perceptions of effort and impaired race performance—despite unchanged physiological metrics. These findings demand that coaches, psychologists, and other members of the performance team consider the psychological load sustained by their athletes inside and outside their sports environment to prevent mental fatigue. Training and cognitive strategies specifically targeting mental fatigue must also be further developed.

You et al. examined conscious movement control and inhibition, highlighting this shift, revealing that an athlete's tendency to monitor movements consciously—often seen in high-pressure scenarios—can either enhance or hinder performance depending on task demands and mental fatigue. While simple actions may not benefit from overthinking, complex or reactive skills (like stopping a movement mid-action) may rely on conscious control, particularly when cognitive resources are depleted. These findings underscore the importance of individualized training approaches, blending implicit learning with strategic focus cues and preparing athletes to perform under mental fatigue. As sports enter the cognitive era, optimizing the interplay between automaticity and conscious control will be key to unlocking peak performance.

## Beyond open vs. closed skills: cognitive demands matter

Recent findings challenge the assumption that open-skill sports (e.g., football) inherently sharpen executive functions more than closed-skill sports (e.g., golf) is being challenged. Li et al. found that 16 weeks of golf training improved inhibitory control comparably to football, suggesting that cognitive demand, not just sport classification, drives neurocognitive benefits. It expands the horizon for using closed-skill disciplines in cognitive training.

## Motivation and motor learning: the power of choice and reward

Quan et al. close the loop with a compelling insight: reward and autonomy (voluntary choice) independently boost motor skill acquisition. Though their effects fade in retention, their synergy during training could optimize skill refinement—a principle applicable from rehabilitation to elite coaching. Supporting this, a study on motivational imagery Habib et al. demonstrated its effectiveness: when tested on 20 female undergraduates, motivational imagery significantly increased intrinsic motivation and physical activity over 12 weeks compared to a general physical activity imagery intervention, reinforcing motivational imagery as a cognitive-behavioral strategy to enhance engagement in exercise.

## Toward a cognitive revolution in sports science

Collectively, these studies signal a transformative era where cognitive abilities such as strength, speed, and endurance are recognized as critical to sports performance. However, these studies highlight the complex interaction between cognitive, perceptual, emotional, and motivational factors. Therefore, it is important to consider the whole athlete's psychology as well as the underlying neurobiology. Future research must further integrate psychology and neurobiology and fill gaps, e.g., longitudinal studies of cognitive skills development and individualized mental fatigue monitoring and management. One conclusion is becoming clear: the mind/brain is the next frontier of performance enhancement.

Cognitive training is just as important as physical training in sports. We suggest that future studies focus on mental coaching that combines brain science with athletic training to help athletes perform at their best, both mentally and physically.

## Implications for practice

- Integrate mindfulness and cognitive training into regimens to reduce anxiety and sharpen decision-making.
- Monitor and mitigate mental fatigue with tailored recovery protocols.
- Prioritize sport-specific perceptual-cognitive drills to accelerate expertise.
- Leverage autonomy and reward structures to optimize motor learning.

